# Research capacity and culture in an Australian metropolitan public mental health service: scoping the skills and experience of social workers and occupational therapists

**DOI:** 10.1186/s12909-022-03936-0

**Published:** 2022-12-14

**Authors:** Christine Migliorini, Caitlin McDowell, Megan Turville, JoAnne Bevilacqua, Carol Harvey

**Affiliations:** 1 NorthWestern Mental Health, Royal Melbourne Hospital, Parkville, Australia; 2 Centre for Youth Mental Health, The University of Melbourne, Melbourne, Australia; 3 Psychosocial Research Centre, Department of Psychiatry, The University of Melbourne, Melbourne, Australia

**Keywords:** Evidence-based Practice, Mental health, Health workforce, Allied health personnel, Organisational culture, Research capacity, Research culture

## Abstract

**Background:**

Investment in a clinical research culture appears to be associated with benefits for consumers, staff, and overall organisational performance. The validated 55-item Research Capacity and Culture (RCC) tool was developed specifically to gauge the research capacity and culture of health professionals and workplace settings within which they work. Results of some individual studies suggest that professional discipline and workplace setting may impact RCC results however it has never been used in a dedicated public mental health setting. Therefore, this study will explore the research capacity and culture of allied *mental health* clinicians (Part 1). Another aim is to explore potential connections between workplace settings, locations and disciplines based on published RCC-based data to help signpost potential impediments to service improvements (Part 2).

**Methods:**

Part 1: An RCC-based online survey canvased Australian Social Workers and Occupational Therapists (*n* = 59) based in a metropolitan public mental health service. Non-parametric analyses explored links between research-related experience and participant characteristics.

Part 2: Comparative analyses explored the potential influence of workplace settings and professional disciplines on published RCC results.

**Results:**

Part 1: Overall, the research capacity and experiences of mental health Social Workers and Occupational Therapists seemed modest. Discipline was statistically associated with level of research-activity experience, weighted towards occupational therapy; demographic characteristics were not. Only two items in the RCC were rated high; many more items were rated low.

Part 2: Published studies exploration found no link between RCC ratings and workplace location, setting, or professional discipline. Sampling biases and use of modified, non-validated RCC versions likely impacted the results.

**Conclusions:**

Allied mental health clinicians may not be sufficiently experienced, knowledgeable, or confident with a range of research-related activities given the emphasis on workforce research capability in policy and practice nowadays. This may be commonplace across health-based organisations. We recommend the systematic implementation of research training programs in (mental) health services, and a ‘whole-of-service levels’ approach be used i.e., transform policy, culture and leadership as well as provide practical resources with individual training. Potential benefits include a positive impact on organisation functioning, clinicians’ confidence and practice, and improved consumer outcomes.

**Supplementary Information:**

The online version contains supplementary material available at 10.1186/s12909-022-03936-0.

## Background

Disciplines such as occupational therapy and social work report being under pressure to deliver clinically relevant, cost-effective and knowledge (or research) informed practices [1, 2]. Allied health clinicians develop basic research-skills training during their undergraduate course, yet it is relatively unknown how these skills develop within the workforce [3]. Investment in a clinical research culture appears to be associated with benefits for consumers, staff and overall organisational performance [4]. Hardy [4] defines research culture as “a culture in which the application of evidence is valued, clinicians are encouraged to participate in research-related activities, opportunities are available for staff to acquire skills in research and evidence-based practice, research achievements are recognised and there is an investment of resources in research activity (p45)”.

Despite interest in research, allied health clinicians report many barriers including heavy workloads, staff turn-over and low staffing levels; lack of organisational resources and infrastructure; lack of own skills and knowledge; and low support from managers and colleagues [5–7]. These impediments seem to lie across individuals, practice-based teams, and organisations/services. Consequently, to remedy these barriers it is important to gauge the level of research-related skills, knowledge and experience within individuals, the capacity of the organisation infrastructure to support research, and the organisation’s culture or appetite for encouraging clinician-driven research.

A tool that was recently developed in Australia is the Research Capacity and Culture survey (RCC) [8]. The RCC was specifically developed to identify and understand the research capacity and culture of the health workforce and quantitatively measures the research capacity and culture across three levels: organisation, team and individual. The RCC was validated with excellent results [8] and has since been widely used in general medical health settings both in Australia (e.g. [9–12]) and overseas (e.g. [13, 14]).

Based on RCC results, workplace settings have been linked to the research capacity and confidence of clinicians. For example, Williams, Lazzarini et al. [15] found statistically significant differences in the self-rated research skills of Australian podiatrists across each of the RCC Individual domain items, with the breakdown from highest to lowest being non-clinical roles (managers, educators, and academics), public hospital, public community, private practice. Williams, Miyaki et al. [9] found that workplace location made a unique contribution to the variance in all RCC domain items rated by Victorian allied health clinicians, according to whether the health service was located in a metropolitan or regional area, weighted in favour of metropolitan area.

Similarly, professional discipline has been linked to the research capacity and confidence of clinicians. For example, Lee [11] found that individual research capacity differs according to discipline with both allied health staff and nursing staff being less confident across a range of research-based activities compared to medical staff in a Sydney-based general medical setting. Furthermore, two separate studies conducted within the same Queensland-based general health workplace and only two years apart, revealed medical doctors’ RCC scores [16] to be substantially higher than those of allied health professionals [17] indicating medical staff were more confident, skilled and experienced in research-related matters as well as evidently receiving more resources and support from their teams and the organisation as a whole compared to other health disciplines. This suggests that professional discipline may impact research confidence, skills, and perhaps even availability of resources.

While studies that have used the RCC often included mental health clinicians albeit in small proportions within their overall samples, all have been based within general medical settings (typically comprehensive medical service providers), and none were based in a dedicated mental health service setting. Therefore, the aim of this project is to explore the research-related knowledge and skills of allied health clinicians working within a metropolitan public mental health service and the capacity and culture of the organisation, using the RCC tool (Part 1).

There is some evidence that workplace settings and professional discipline may impact the research capacity and culture of clinicians. Therefore, a further aim was to explore the potential connections that workplace setting, location and professional discipline may have with research capacity and culture using the results of this study along with RCC-based data sourced from published studies (Part 2). Undertaking this extended exploration may provide clues about which of those factors, if any, may be linked with higher RCC scores and consequently best practice.

## Methods

### Part 1: within dedicated public mental health setting

An exploratory mixed methods study design was implemented consisting of an online survey and semi-structured interviews. The anonymous online survey consisted of demographic and work-related items, the validated RCC tool [8], eight open-ended questions and a call for volunteers willing to be interviewed. The qualitative results from the open-ended questions and semi-structured interviews will be reported elsewhere. Survey data were collected and managed using REDCap [18] an electronic data capture tool hosted by the Royal Melbourne Hospital Business Intelligence Unit.

#### Workplace setting

This study was conducted in a metropolitan public mental health service in Victoria, Australia. This area-based mental health service provides a comprehensive range of specialist, community, and hospital-based mental health services for youth, adult and aged people who are experiencing, or are at risk of developing a serious mental illness. Services are delivered across a range of locations, including most major hospitals within the service’s area of responsibility, and a range of community-based clinics. In addition, the service delivers several state-wide specialist services including the Neuropsychiatry Unit and an Eating Disorders Program. The project focused on social workers and occupational therapists for pragmatic reasons. Arguably these two MH-based disciplines receive very similar levels of research-related training during their undergraduate years. In addition, occupational therapy had employed a Research Lead (a standalone position dedicated to promoting research projects and knowledge translation activities) for some years while the social work department did not have an equivalent dedicated standalone position until recently.

#### Recruitment method

All social workers and occupational therapists employed within the service received a recruitment email comprising an invitation to participate in the project, an attached plain language statement and a link to the anonymous online survey, via their ‘discipline specific all-staff’ emailing list. The recruitment email was re-sent on a further two occasions within a six-week period during October and November 2020. The inclusion criteria were being a qualified social worker or occupational therapist employed within the mental health service at the time of recruitment, regardless of position/role, hours worked per week or whether employed on a full-time, part-time, contract or casual basis.

#### Participant demographic and discipline-related characteristics

The online survey comprised demographic and discipline related information including gender, age, discipline, highest education level, and years since completed last qualification. Educational level was partitioned into (a) undergraduate only (b) undertaking postgraduate coursework (c) completed postgraduate coursework (d) undertaking postgraduate research, and (e) completed postgraduate research. Time since last qualification was partitioned into 5-year subgroups and a 20-years + subgroup.

#### Participant research-related activity characteristics

Aside from the RCC tool (see below), participants were asked to indicate all the types of research-related activities with which they had been involved, over the last 5-years. They were asked to tick *all* that applied, with the options being (a) project work (b) quality assurance or QA (c) evaluation (d) research, and (e) none of the above; these subcategories were not mutually exclusive except for ‘none of the above’. This item provides evidence about the range or breadth of research-types with which participants had been involved, as well as the number of types within individuals. These qualities were used for descriptive purposes only. While these activities are ordinarily discussed as discrete types of undertakings, they operate on a continuum from quality assurance to original research [19–21]. Moreover, all involve the processes of identifying/appraising literature, identifying levels of risk and the ethical consequence of same, use scientific methodology to collect empirical data, and utilising the results [19–21] and consequently require a similar set of skills. Arguably, project work in mental health services also involve very similar processes i.e., identify a clinical problem then appraise the literature, identify risk, implement program, collect data/evaluate, and utilise results. Therefore, it is reasonable to surmise that a similar set of skills would be needed so for the purposes of this study, all terms were considered equivalent; for the analysis the variable was converted to a dichotomous variable of any research experience and no research experience.

#### Research capacity and culture tool (RCC)

The RCC [8] is a self-report tool with excellent internal consistency with Cronbach alpha coefficients reported across the three domains of Organisation (20 items), Team (20 items) and Individual (15 items): α = 0.95, 0.96 and 0.96, respectively. In this study, the Cronbach alpha coefficients for the domains of Organisation, Team and Individual were α = 0.97, 0.98 and 0.95 respectively, indicating excellent internal consistency and reliability of the scale. Each item can be scored from 1 = Lowest possible skill or success level, to 10 = highest possible skill or success level. Accordingly, the domain score ranges are Organisation 20 to 200, Team 20 to 200, and Individual 15 to150. Median and interquartile range (*IQR*) were calculated for the summary RCC item and domain statistics since they violated the assumption of normal distribution: almost every item was positively skewed, and kurtosis values were below zero. Median average scores were collapsed into three subcategories of low skill/success (scores 1–3.99), moderate skill/success (scores 4–6.99), and high skill/success (scores 7–10), in keeping with previous studies such as Alison [12], Friesen [6], and Matus [17].

##### Within study analyses

Non-parametric analyses (Chi-Square Test of Independence and Fisher’s Exact Probability Test) were used to explore associations between the participant characteristics and background with (any) experience in research-related activities, due to the categorical nature of the variables and the size and distribution of the sample.

To comply with the minimum cell frequency assumption of ≥ 80% of cells should have expected frequencies of ≥ 5 associated with Chi-square test for independence, the following variables were transformed: Age groups were condensed into three groups (20-34yrs, 35-49yrs, 50yrs +); Highest education level groups were condensed into three groups (undergrad only, PG-coursework (undertaking or completed), PG- research (undertaking or completed)); and, Time since last qualification groups were condensed into three groups (0-6yrs, 7-10yrs, 11yrs +).

A Friedman test was performed to determine whether there was a difference in the median scores between Organisation, Team and Individual RCC domains. Post hoc Wilcoxon Signed Rank Tests were performed to determine where the differences lay. Effect sizes (*r*) were calculated with interpretation based on Cohen’s criteria of 0.1 = small effect, 0.3 = medium effect, 0.5 = large effect. Mann–Whitney U Tests were performed to determine if RCC domain results were associated with any research-related experience [22].

### Part 2: across published studies comparative analyses: workplace settings and discipline

An extended exploration was undertaken to look at factors (Workplace setting, professional discipline) that might be associated with higher RCC scores and consequently potential best practice. Three methods to identify relevant studies were used. The first method was to search Google Scholar using the term “research capacity and culture”. Since Google Scholar is less rigorous, more generous in search returns, it was thought likely most if not all studies would be listed. The second method was to repeat this method using Google itself. The third method was to use the online citation analysis tool CitationChaser which searched for citations across the LENS.org database (consisting of PubMed, PubMed Central, CrossRef, Microsoft Academic Graph and CORE), using the DOI of Holden’s 2012 foundation validation article. The first two methods were ‘topic-based” methods. The third method was a citation search based on the foundation article that introduced the RCC.

The inclusion criteria for studies to be included in our analyses were required to be:

- Peer-reviewed
- Based in Australia – since health systems across countries are too dissimilar
- Report RCC sourced data across the three RCC categories of organisation, team and individual – since the objective of Part 2 is to compare RCC results across workplace settings and disciplines
- Report results as medians and IQR – while both means and medians are both types of statistical averages, means should only be used when the distribution of data is normally distributed (i.e., bell shaped distribution) as means are unduly influenced by skewed data and outliers. In contrast, medians are robust measures of central tendency and used for non-normal distributions of data. Consequently, means and medians should not be used within the same comparative analysis

All articles that cited the foundation article published in 2012 up to July 2021 found by CitationChaser confirmed our Google Scholar results. Thirty citations were identified with 20 citations being excluded due to: 5 × citations mentioned RCC but did not include any data [23–27], 1 × citation included RCC domain Team data only [28], 2 × citations were based in educational settings [29, 30]; 5 × citations were based overseas [13, 14, 29–31], 10 × citations used means and standard deviations [6, 28, 32–39], and 2 × citations were not peer reviewed [37, 39] [NB: these categories are not mutually exclusive]. See Table 1 for the final list of 10 included studies and full breakdown of study sample characteristics.

**Table 1:** Descriptive features of samples used within peer-reviewed published studies that used the RCC (abridged version described)

Author, date	Sample size, discipline breakdown, study setting (as published)	Summed Org’n (18–180)	Summed Team (19–190)	Summed Individual (14–140)
Current study	59 **Occupational Therapy** and **Social Work** **Victorian metropolitan public mental health service**	84.0	64.0	47.0
Brandenburg (2021) [16]	124 **Medical doctors:** Consultants (72.5%), Junior doctor (12.9%), Registrar (12.1%), Not specified (3%) **Gold Coast Health**	92.0	106.0	75.5
Lee (2020) [11]	393 health professionals: **Medical** n = 72 (18%): Clinician (84.5%), Management/Executive (5.6%), Teaching/Research (9.9%); **Nursing** n = 139 (35%): Clinician (53.3%, Management/Executive (17.5%), Teaching/Research (29.2%); **Allied Health** n = 182 (46.3%): Clinician (61.2%), Management/Executive (19.1%), Teaching/Research (19.7%) [21% of total sample = teaching/research; 6% of total sample = management/executive] **Western Sydney Local Health District**	Medical 93.0	Medical 111.5	Medical 93.0
		Nursing 93.5	Nursing 73.0	Nursing 63.5
		Allied Health 79.5	Allied Health 68.0	Allied Health 74.0
Matus (2021) [7]	320 **Allied Health professionals**: Physiotherapy (31%), Occupational Therapy (26%), Social Work (16%), Speech pathology (7%), Clinical Psychology/Clinical Neuropsychology (5%), Dietetics (4%), Other (4%), Allied health assistant (2%), Not disclosed (1.5%) [32% of sample reported research to be part of their role] **South Metro Health Service in WA**	96.0	79.5	52.5
Matus (2019) [17]	302 **Allied health professionals** and **allied health assistants**: Occupational Therapy (19%), Pharmacy (13%), Physiotherapy (12%), Psychology (11%), Speech pathology (10%), Nutrition & Dietetics (9%), Social Work (8%), Medical imaging (4%), Allied health assistant (3%), Other/prefer not to say (3%), Public Health (3%) Oral health (2%), Clinical measurements (2%), Orthoptics (< 1%), Welfare (< 1%) **Gold Coast Health**	122.0	118.0	65.0
Alison (2017) [12]	276 **allied health professionals**: Physiotherapy (30%), Occupational Therapy (19%), Nutrition & Dietetics (17%), Pharmacy (14%), Social Work (8%), Radiography (4%), Psychology (3%), Others (4%), Speech pathology (1%), Podiatry (1%) **Sydney Local Health District**	114.0	107.0	68.0
Gill (2019) [10]	776 Health organisation staff: Nursing (33.6%), Medical (16.4%), Allied Health (26.4%), Mental health (5.4%), Medical science (2.7%), Administration and clerical (7.9%), Corporate (3.7%), Other (3.9%) 8 of the 11 health service partners in the **Western Alliance** located in **rural/regional Southwest Victoria**	96.0	95.0	70.0
Williams, Lazzarini (2015) [15]	233 **Podiatrists** **Across Australia in private (57%), public (31%) and non-clinical (12%) settings**	87.0	80.5	49.0
Lazzarini (2013)^a^ [40]	33 P**odiatrists** **QLD Health** in 2012	133.0	107.5	68.0
Williams, Miyazaki (2015) [9]	539 **Allied Health disciplines** and all grades represented: Physiotherapy (27%), Occupational Therapy (16%), Dietetics (12%), Social Work (9%), Speech pathology (8%), Not specified (5%), Psychology (4%), Radiography (4%), Podiatry (3%), Audiology (2%), Music therapy (2%), Orthotics & Prosthetics (2%), Pharmacy (2%), Exercise physiology (1%), Oral Health (not dentistry) (1%), Radiation therapy (1%), Sonography (1%), Drug and alcohol (< 1%), Pathology (< 1%), Play therapy (< 1%), Orthoptist (< 1%) **Victorian public health sector:** metropolitan (83%)	110.0	102.0	65.0
Holden (2012) [8, 34]	134 **Allied health professionals**: Allied health assistants (2%), Dieticians (7%), Occupational Therapists (18%), Physiotherapists (22%), Speech pathologists/Audiologists (7%), Social Workers (15%), Psychologists (4%), Other (10%), Missing (13%) **QLD Health**	86.5	75.0	52.0

Settings and discipline were explored in two ways. The first was a graphical exploration whereby the median item scores from each study were collapsed into three subcategories of low skill/success (scores 1–3.99), moderate skill/success (scores 4–6.99), and high skill/success (scores 7–10), in keeping with previous studies such as Alison -[12], Friesen [6], and Matus [17]. The number of items located within each subcategory within each domain were then summed and the relative distribution displayed in graphs for visual inspection.

The second way was to apply three comparative bivariate statistical analyses on the reported RCC outcomes using the summed RCC domain median item scores from each study as the dependent variable with the independent variables being professional discipline (specified discipline or mixed disciplines), workplace setting (single service/organisation or multiple organisations), and location (metro, regional/rural, state/national). Please note, Lee’s study [11] reported RCC results as three discrete professional discipline types: doctors, nurses, and allied health professionals. For the comparator analyses where the independent variable was professional discipline, these groups were treated as discrete independent variables. For the comparator analyses where the independent variable was workplace setting, the mean of these three groups was used since each group was sampled from the same setting.

The 10 included publications were Australian studies that reported median and IQR per item per RCC domain. Excluded were 13 studies that reported means and standard deviations (*n* = 10) [6, 28, 32, 33, 35, 36, 38, 39], were set outside of Australia (*n* = 3) [13, 14, 31], were not peer-reviewed [37, 39] and/or did not report RCC results for each domain [34].

It is important to note that for comparative purposes, two items (#4 and #20) from the domain Organisation, and one item from each of the domains Team (#30) and Individual (#54) were omitted from Lazzarini [40], Holden [8] and the current study results, to match the abridged versions of the RCC used by the other comparator studies. Consequently, the range of potential summed scores became: Organisation = 18–180, Team = 19–190, and Individual = 14–140.

The data was analysed using IBM SPSS v26 and MS Excel 2013, and the Free Statistics Calculator v4 (see Free Statistics Calculators—Home (danielsoper.com) for the Fisher’s Exact Probability Test with Freeman-Halton extension for the 2 × 3 tables. *P*-values < 0.05 were considered statistically significant. Effect sizes were calculated for statistically significant results. Cohen’s recommended interpretation of the phi coefficient effect size is as follows: 0.10 for small effect, 0.30 for medium effect and 0.50 for large effect [22].

All applicable institutional and governmental regulations concerning the ethical use of human volunteers and in accordance with the Declaration of Helsinki, were followed during the course of this research. Each participant received a plain language statement and opportunity to ask questions about the study. Informed consent was deemed given with the voluntary completion of the survey. This project received full ethics approval from the Melbourne Health Ethics Committee: HREC/64816/MH-2020.

## Results

The overarching aim of this project was to broadly explore the research-related knowledge, skills and experience of allied health staff working within a large Australian metropolitan public mental health service and the research-related capacity and culture of the organisation. A further aim was to consider the potential connections that workplace settings and professional discipline may have with research-related capacity and culture using the results of this study along with the RCC -based data sourced from published studies. Included studies were all peer-reviewed, Australian-based studies that reported median and interquartile range per item per RCC domain.

### Part 1: participant characteristics and background

#### Survey participants

Overall, 59 social workers and occupational therapists responded to the survey invitation, representing an approximate 22% response rate. The most common participant characteristics were female, aged 30-39yrs, had completed postgraduate coursework within the last two years, and was a Social Worker. See Table 2. Most participants (58%) had experience with project work and to a lesser extent evaluation (39%) while 32% of participants indicated that they had no experience with any of the research-related activity types.

**Table 2:** Demographic characteristics of Survey completers (*N* = 59)

Variable	Subcategory	Count (percent)^a^
Discipline	Social Work	35 (59%)
	Occupational Therapy	24 (41%)
Gender	Female	49 (83%)
	Male	10 (17%)
Age	20-29yrs	10 (17%)
	30-39yrs	21 (36%)
	40-49yrs	12 (21%)
	50-59yrs	14 (24%)
	60yrs +	1 (2%)
Highest Education	Undergraduate only	17 (29%)
	Undertaking postgraduate coursework	5 (9%)
	Completed postgraduate coursework	29 (49%)
	Undertaking postgraduate research	– (0%)
	Completed postgraduate research	8 (14%)
Time since last qualification	0-4yrs	22 (37%)
	5-10yrs	15 (26%)
	11-14yrs	8 (14%)
	15-19yrs	7 (12%)
	20yrs +	7 (12%)
Has research-related activity experience with^b^	Project Work	34 (58%)
	QA	15 (25%)
	Evaluation	23 (39%)
	Research	17 (29%)
	None of the above	19 (32%)
Breadth of experience^c^	No research-related experience	19 (32%)
	1 activity type	16 (27%)
	2 activity types	6 (10%)
	3 activity types	11 (19%)
	4 activity types	7 (12%)

^a^Percentages will not always add to 100% due to rounding

^b^Percentages will not always add to 100% due to rounding

^c^Responses to subcategories were not mutually exclusive except for ‘None of the above’

##### Within study analyses

Associations between the categorical independent variables (Time since completed last qualification, Professional Discipline, Age group, and Gender) with any level of research-related activity experience were explored. Statistical non-significance was found for Time since completed last qualification (*p* = 0.53), Age group (*p* = 0.78), and Gender (*p* = 0.27). A statistically significant difference was found for Professional Discipline (*p* = 0.048) with a small to medium effect size (*phi* = 0.28) weighted towards occupational therapists more likely to have research-related experience.

See supplementary material for more detail.

### Research capacity and culture tool (RCC)

#### RCC—Organisation

Within the domain Organisation, the most common median scores fell within the medium category. The median score for three items relating to organisation-provided resources was low: (#1) *Provides resources to support staff research training*; (#2) *Provides funds, equipment or admin to support research training*; and (12) *Provides software programs for analyzing research data*. In contrast, the median score for two items relating to the way clinicians are expected to practice was categorised as high: (#10) *Promotes clinical practice based on evidence*; and #20 *Requires ethics approval for research activities*. See Table 3.

**Table 3:** Median and Interquartile (IQR) range, and relative category per item in RCC domain Organisation

*Does your organisation*	*Median (IQR)*	*Median category per item*
1. Provides resources to support staff research training	3 (2–5)	Low
2. Provides funds, equipment or admin to support research training	3 (2–5)	Low
3. Has a plan or policy for research development	5 (3–5)	Moderate
4. Provides access to literature search and article retrieval^a^	5 (3–7)	Moderate
5. Has senior managers that support research	5 (3–7)	Moderate
6. Ensures staff career pathways are available in research	4 (2–5)	Moderate
7. Ensures organization planning is guided by evidence	6 (4–7)	Moderate
8. Has consumers involved in research	5 (3–7)	Moderate
9. Accesses external funding for research	5 (3–6)	Moderate
10. Promotes clinical practice based on evidence	7 (5–8)	High
11. Encourages research activities relevant to practice	5 (3–7)	Moderate
12. Provides software programs for analysing research data	3 (2–5)	Low
13. Has mechanisms to monitor research quality	4 (3–6)	Moderate
14. Provides experts for research advice	5 (3–7)	Moderate
15. Supports a multi-disciplinary approach to research	4 (3–6)	Moderate
16. Provides forums or bulletins to present research findings	5 (3–7)	Moderate
17. Engages external partners (e.g. universities) in research	5 (3–7)	Moderate
18. Supports applications for research scholarships or degrees	5 (2–6)	Moderate
19. Supports the peer-reviewed publication of research	5 (3–6)	Moderate
20. Requires ethics approval for research activities^a^	7 (5–9)	High

^a^denotes items not included in the abridged versions of the RCC used in the published studies

#### RCC -Team

Within the domain Team, the most common median score fell within the low category. No team items could be classified as high. Seven team items could be classified as medium, tending towards the lower end of medium. Participants indicated that they had some confidence/success in team leaders who support research (#25), that consumer involvement in research is encouraged (#28), that experts are accessible for research advice (#34), and that disseminating research results with others at research forums/seminars is supported (#35). In contrast, items that could be linked to the provision of resources (#21), equipment & funds (#22) and other ‘doing’ activities such as applying for funds (#29), conducting research (#31), accessing literature (#30) were rated low. See Table 4.

**Table 4:** Median and interquartile (IQR) range and relative category per item in RCC domain Team

*Does your team*	*Median (IQR)*	*Median category per item*
21. Provides resources to support staff research training	3 (2–5)	Low
22. Provides funds, equipment or administration to support research activities	3 (2–5)	Low
23. Does team level planning for research development	3 (1–5)	Low
24. Ensures staff involvement in developing that plan	3 (1–5)	Low
25. Has team leaders that support research	4 (2–6)	Moderate
26. Provides opportunities to get involved in research	3 (2–5)	Low
27. Does planning that is guided by evidence	5 (3–6)	Moderate
28. Has consumer involvement in research activities or planning	4 (2–6)	Moderate
29. Has applied for external funding for research	3 (1–5)	Low
30. Provides access to literature searching and article retrieval^a^	3 (2–5)	Low
31. Conducts research activities relevant to practice	3 (2–5)	Low
32. Supports applications for research scholarships or degrees	3 (1–6)	Low
33. Has mechanisms to monitor research quality	3 (1–5)	Low
34. Provides experts accessible for research advice	4 (2–5)	Moderate
35. Disseminates research results at research forums or seminars	4 (2–6)	Moderate
36. Supports a multi-disciplinary approach to research	4 (2–6)	Moderate
37. Has incentives and support for mentoring activities	3 (1–5)	Low
38. Has external partners (e.g. universities) engage in research	3 (2–5)	Low
39. Supports peer-reviewed publication of research	4 (1–5)	Moderate
40. Provides software to support research activities	2 (1–4)	Low

^a^denotes item not included in the abridged versions of the RCC used in the published studies

#### RCC – Individual

Within the domain Individual, the most common median score fell within the low category. Six items could be classified as medium: finding (#41), reviewing (#42) & integrating (#54) the literature, designing questionnaires (#47), collecting data (#48), and analysing qualitive research data (#50). However, no item could be classified as high. See Table 5

**Table 5:** Median and interquartile (IQR) range and relative category per item in RCC domain Individual

*Individual*	*Median (IQR)*	*Median category per item*
41. Finds relevant literature	6 (4–7)	Moderate
42. Critically reviews the literature	5 (3–7)	Moderate
43. Uses a computer referencing system (e.g., Endnote)	3 (1–5)	Low
44. Writes a research protocol	3 (1–5)	Low
45. Secures research funding	2 (1–4)	Low
46. Submits an ethics application	2 (1–5)	Low
47. Designs questionnaires	5 (2–7)	Moderate
48. Collects data, e.g., surveys, interviews	5 (2–7)	Moderate
49. Uses computer data management systems	3 (1–5)	Low
50. Analyses qualitative research data	4 (1–6)	Moderate
51. Analyses quantitative research data	3 (1–6)	Low
52. Writes a research report	2 (1–6)	Low
53. Writes for publication in peer-reviewed journals	2 (1–4)	Low
54. Integrates research findings into practice^a^	5 (2–7)	Moderate
55. Provides advice to less experienced researchers	2 (1–5)	Low

^a^denotes item not included in the abridged versions of the RCC used in the published studies

#### Differences between RCC domains within the mental health sample

The overall median and interquartile scores for each domain were Organisation *Md* = 5.0 (IQR: 3.0–6.0), Team *Md* = 3.0 (IQR: 1.0–5.0), and Individual *Md* = 3.0 (IQR:1.0–5.0). The results of the Friedman Test indicated that there was a statistically significant difference in RCC skills/success levels across the three domains (Organisation, Team, Individual, (*χ2* (2, 59) = 21.8, *p* < 0.001). Three post hoc Wilcoxon Signed Rank Tests (using Bonferroni adjusted alpha values to control for Type 1 error: 0.05/3 = 0.017) compared Organisation with Team, Organisation with Individual, and Team with Individual. There was a statistically significant higher endorsement of Organisation skill/success levels compared to both Team (*z* = -4.7, *p* < 0.0005) with a medium effect size (*r* = 0.43) and Individual (*z* = -3.4, *p* = 0.001) with a medium effect size (*r* = 0.32). There was a non-significant difference between Team and Individual (*p* = 0.88, *r* = 0.01).

Mann–Whitney U Tests revealed no significant differences in overall RCC domains Organisation or Teams in participants with and without research-related experience. There was a statistically significant difference in skill/success levels in RCC domain Individual between participants with research experience and those without, weighted in favour of with experience (*p* = 0.04) however, the effect size was small (*r* = 0.27). See supplementary file.

### Part 2: across published studies comparative analyses: workplace settings and professional discipline

**Fig. 1:**
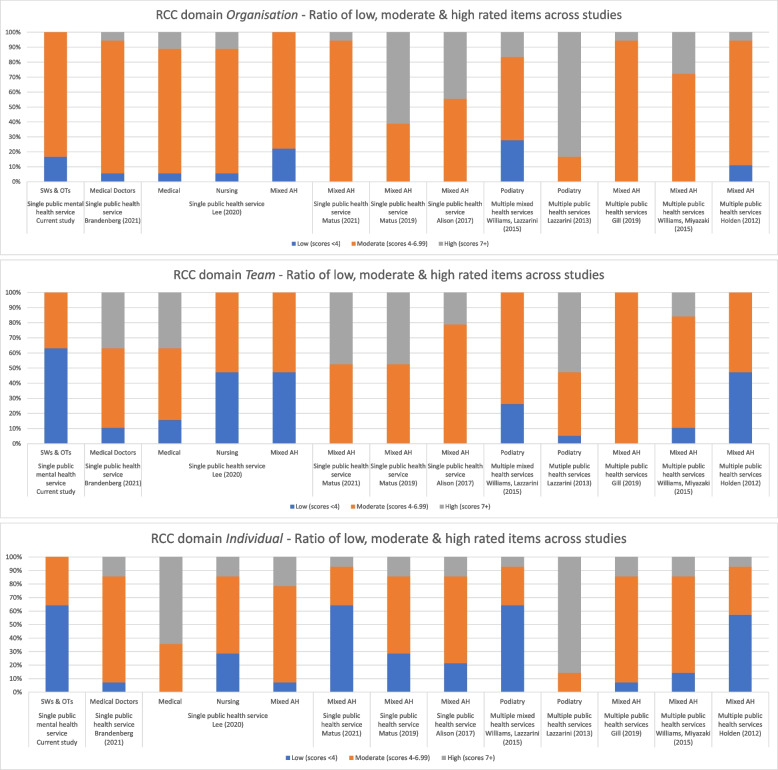
Graphical display of relative groupings of RCC items across published studies

Graphical display of the relative groupings of the RCC item ratings reveals seemingly strong differences between studies and therefore ratings as reported by the individuals within each study sample. Overall, the proportion of items within the poor category (Organisation = 7%, Team = 21%, Individual = 28%) seemed to increase the more proximal the domain is to the individual. The proportion of items within the high category (Organisation = 21%, Team = 20%, Individual = 21%) seemed to be relatively stable across the domains. However, the most common rating within each of the domains was moderate (Organisation = 71%, Team = 59%, Individual = 51%). See Fig. 1.

Comparative analyses were possible to explore whether the workplace setting (single health service or multiple health services) or location (metro, regional/rural, state/national), or professional discipline (discipline specific or mixed disciplines) influenced RCC domain scores. Item medians per RCC domain were summed within each study thus offering an overall representation of the perceived research capacity and culture in the various health settings investigated by the selected published studies. Two independent samples *t*-tests and one ANOVA were conducted. Neither workplace settings (single service or multiple services: Organisation *p* = 0.55; Team *p* = 0.91; Individual *p* = 0.37) nor the discipline (specific discipline or mixed discipline: Organisation *p* = 0.0.71; Team *p* = 0.88; Individual *p* = 0.72), nor workplace location (metro, regional/rural, state/national: Organisation *p* = 0.77; Team *p* = 0.23; Individual *p* = 0.32) were statistically significant across the domains. See supplementary material.

## Discussion

To the best of our knowledge, this is the first study to use the RCC in a public mental health service in Australia or internationally. This discussion will begin by reflecting on the within mental health service results (Part 1) followed by the across studies results (Part 2). Overall, the reported research capacity and experiences of the allied mental health clinicians seem modest but not unlike some earlier studies e.g. Holden et al. [8]. Only two items across all the RCC items were rated highly; many more items were rated low. The domain Organisation seemed to elicit the most homogenous range of responses, having a somewhat normal distribution; the highest number of median item responses fell within the moderate range. Interestingly, it was two Organisation domain items that related to how clinicians are expected to practice (promoting clinical practice based on evidence and requiring ethics approval for research activities) that received the highest rating from participants.

There was more variability in the Team related items both within the current study based in a mental health service as well as other health services represented in the published studies e.g. Holden et al. [8], and Lee et al. [11]; clearly, some teams work better than others. This could be because there are many different teams within a mental health service. Leadership style has also been linked with team functioning e.g., see Corrigan et al. [41] and Hitch et al. [42]. Participants came from several teams within the mental health service and likely experienced a range of different leadership styles though this was beyond the remit of this study to investigate. Our results could be evidence that obstacles are in place that prevent the more positive overall organisational culture from filtering down adequately. If this was the case, then it would be reasonable to surmise that low ratings would increase as items became more closely related to the individual and consequently more distal from the organisation as a whole and indeed, that was found. An alternative explanation could be the survey terminology itself. Team can mean different things to different people, like the definitions of family. Within public mental health services, a team can be a discipline specific team within a service such as a team of Social Workers or Occupational Therapists or a multidisciplinary collaborative outreach team that could comprise social workers, occupational therapists, nurses, psychologists, medical staff and/or lived experience consultants. Teams can be based in the community or within an acute inpatient setting. In recognition of these differences, this study deliberately did not provide a definition of team, leaving it to the individual to decide where they saw themselves and perhaps, unintentionally allowed bias to creep into the results. A further possibility is that it may not be seen as the role of small units such as teams, to support research (e.g., many teams would not have the capacity to provide funds and if particular supports are provided by the organisation, then perhaps, need not be duplicated by individual teams). Alternatively, these results could simply reflect characteristics of a modest sized sample.

Most of the Individual items were classified as low suggesting that participants rated their research-related skills and knowledge as low. This may be expected when looking at the breadth of their research-related activity experience but perhaps not if one considers the level of post-graduate education represented in the cohort. Participants did feel moderately confident in finding, critically reviewing, and integrating the literature into their practice. Arguably these were the items that are the most amendable/transferable to everyday practice. In contrast, it was the items more closely associated with doing research, evaluation, and quality assurance projects [43–45] such as designing a study, submitting an ethics application, and writing for publication, which were reported low by the participants. Nevertheless, these results are in keeping with earlier studies, revealing this to be a common enough dilemma for allied health clinicians across various health settings (e.g. [7, 8, 12]), possibly reflecting an historical lack of research training and mentoring in the workplace [12].

Exploration of participants’ personal and professional characteristics revealed occupational therapists’ participation in research-related activities in the last five years was significantly higher than social workers however this was not unexpected. The service has a muddled history with long term discontinuities in research support whether occupational therapy or social work, over the years. However, since 2014, the service has employed part/time, an occupational therapist Research Lead with an increase in the quantity of quality assurance projects and knowledge translation activities able to be reported within a couple of years [46]. There has not been an equivalent dedicated standalone position within the social work department until recently and even then, with a smaller time fraction. The potential benefit of employing a discipline research lead is in keeping with the findings of Williams, Miyasaki et al. [9] and Wenke et al. [47], with both studies finding that employing a Research Lead was beneficial to the research capacity, skills and culture of allied health clinicians in other healthcare settings.

It is worth noting that more than half of the participants reported either no experience with research-related activities or experience with only one type, most commonly project work. The lack of experience was not statistically linked to age or time since participants completed their last qualification. As a rule, we are unlikely to participate in activities in which we have no confidence or are not viewed as part of our everyday remit. Fortunately, there is growing empirical evidence that the implementation of research training programs substantially improves confidence, engagement and practice that positively impact on organisational functioning and consumer care (e.g. [4, 48]). Our findings suggest that a systematic implementation of research training programs is needed, with the potential to positively impact organisation functioning, clinicians’ confidence and practice, and consumer outcomes.

### Part 2: across studies commentary

It is worth remembering that comparative research methods underpin discovery, help detect associations and causes that enable theories to be constructed, and deepens our understanding; in short, comparative research methods underpins knowledge development and consequently guides best practice. A further aim of our study was to consider the potential influences that workplace setting, location and professional discipline may have on research capacity and culture using the results of this study along with RCC-based data sourced from published studies. It was thought that this extended exploration may provide clues about which of those factors, if any, may be linked with higher RCC scores and consequently best practice.

There was a variety of distribution patterns across the published studies however the results from the comparative analyses were not significant; neither workplace location, workplace setting, nor professional discipline were statistically linked with RCC ratings. In contrast, both studies led by Williams [9, 15] suggest workplace setting and location does make a difference to the research capacity and culture experienced by clinicians with a greater capacity found in metropolitan vs non-metropolitan locations, and greater capacity in public sector vs private practice. Similarly, the studies by Brandenburg [16] Matus [17] and Lee [11] suggest that professional discipline can make a meaningful difference to the perceived research capacity and culture of the organisation as a whole, the team within which one is based, as well as differing capacities across disciplines, .e.g., medical staff reported greater personal capacity than allied health staff who in turn reported greater personal capacity than nursing staff [11].

Two broad reasons could account for these inconsistencies, (a) Bias and (b) Sampling methods. (a) RCC versions: Our study used the original version of the RCC which meant it included the full complement of items within each domain while all, but the original published studies used abridged versions, though, for the between studies exploration, we omitted the same items for equivalence. Bias may have been introduced with the addition of an unsure/unknown option used by roughly 40% of the published studies (e.g. [7, 12, 16, 17]) but not present in the original version. It is possible that participants in those studies may have nominated usure/unknown rather than rating their workplace organisation and team within the low range thus potentially biasing those study results.

Bias may have been introduced with the variety of abridged RCC versions that have been used. While all publications cite Holden’s validation study that published the original wording, number of items and with forced responses [8], none had used the original version of the tool. Some of the changes have included relatively minor word changes such as changing the tense of verbs – *finds* becomes *finding* (e.g. [9]). Other changes have a more qualitative distinction potentially changing the intent of items. For example, *Provides experts for research advice* became *has identified experts accessible for research advice* (e.g. [15]) that further morphed into *arranges experts to give research advice* [7] by yet another investigator team. Changes made to an instrument should be acknowledged and well-considered. Further validation is required when it is deemed necessary to make such changes.

(b) Sampling methods: Selection bias that may have been introduced by the sampling method used in the published studies. It is unclear what the benefit would be to include corporate and clerical staff along with clinical health professionals in a single sample (e.g. [11]); similarly including clinical health professionals for whom research is a prescribed part of their role together with others where it is not, or including staff such as corporate and clerical staff, and/or allied health or welfare assistants who do not require tertiary education to fulfil their role and therefore would lack research-related training (e.g. [7]). Computing summary outcomes based on such heterogeneous samples is likely to undermine both relevance and generalisability in the results.

Nonetheless, there were limitations to our study too. All the participants in the current study were clinicians who volunteered to complete the survey. While there is no direct evidence available, it is reasonable to assume that survey participants were more interested in research-related activities compared to non-responders. There is no reason why all clinicians must be involved in research, so they need not score highly in the experience-based items within the RCC domain Individual. However, given that survey respondents may have been skewed to those interested in research then it is problematic that so many had little to no experience. All the investigators in our study were also employed by the organisation at the time of the study however we were under no obligation to find any particular result. Our sample size was relatively small resulting in modest power to find statistically meaningful outcomes however our research focus was both explorative and descriptive. Our comparative exploration across published studies was restricted to Australian-based studies that reported RCC outcomes as median and IQR per item per RCC domain. Excluded were studies that reported RCC outcomes in means and standard deviations, studies conducted outside of Australia and/or not peer reviewed. However interestingly, those other studies also made changes to the RCC tool that would make comparative analyses challenging. It would have been interesting to compare clinicians employed in a dedicated mental health setting to those based in general medical settings however this was found not to be a statistically viable option.

Notwithstanding the above, there were strengths to our study. To the best of our knowledge our study was the first study to use the RCC tool exclusively in a public mental health setting. We used the RCC tool in its original validated form enhancing rigor in our results. Our survey was limited to two disciplines thus minimising potential sampling biases. Our survey was both anonymous and used the original forced response methodology thus arguably, minimising potential response bias.

In conclusion, our study provides evidence that allied mental health clinicians based in public mental health settings in Australia may not be sufficiently experienced, knowledgeable, or confident with a range of research-related activities given the emphasis on workforce research capability in policy and practice nowadays. Clinicians had moderate confidence in their ability to find, critically review, and integrate the literature into their practice. However, they were substantially less knowledgeable, experienced, and confident with other research-related activities such as analysing qualitative or quantitative data, designing a study, or submitting an ethics application. Limited comparative analysis to explore the potential link between workplace setting, workplace location and professional discipline, with research capacity and culture was not statistically significant however drilling down into the published studies revealed several methodological biases that may have affected results. Clearly there is much work to be done across the levels of organisation, team, and individual to increase allied mental health clinicians’ skills, knowledge, confidence, and resources connected to research-related activities in health services including mental health services. There is growing empirical evidence that the employment of discipline research leads and the implementation of research training programs positively impacts organisation functioning, clinicians’ confidence and practice, and consumer outcomes. For these reasons, the following recommendations can be made

- Health services, including mental health services, need to take a ‘whole of service levels’ approach to cultivate their research culture/ethos i.e., need to address each level of the organisation (organisation as a whole, teams and individuals) applying different strategies to the different levels
- Health services, including mental health services, need to implement quality training programs that focus on research-related activities
- Health services, including mental health services, need to recognise that some disciplines may require more active support to conduct research-related activities

## Supplementary Information

**Additional file 1.**

## Data Availability

The datasets used and analysed during the current study are available from the corresponding author on reasonable request.

## Abbreviations

RCC: Research Capacity and Culture survey
QA: Quality Assurance
Md: Median
IQR: Interquartile range
