# Supplementary material for: Research capacity and culture in an Australian metropolitan public mental health service: scoping the skills and experience of social workers and occupational therapists
Source: BMC Med Educ. 2022 Dec 14;22:864. doi: 10.1186/s12909-022-03936-0 (PMC9749178; doi:10.1186/s12909-022-03936-0)
Supplement: Supplementary file 1 — Additional file 1. [file 12909_2022_3936_MOESM1_ESM.pdf]

Supplementary Material

Part 1: Within Dedicated Public Mental Health Setting

Time since Education qualification

A Fisher's Exact Probability Test with Freeman-Halton extension (2-tailed) indicated a statistically significant difference between any research-related activity experience and time since last education qualification completed, p = .053.

Table S1 Contingency table displaying distribution of participants according to experience with research-related activities and time since completing last educational qualification

	Time since completed last qualification			
	<5yrs	5-10yrs	11yrs+	Total
Experience	13	8	19	40
No experience	9	7	3	19
Total	22	15	22	59

Age

A Fisher's Exact Probability Test with Freeman-Halton extension (2-tailed) indicated a non-significant difference in any research-related experience according to age group, p = .078

Table S2 Contingency table displaying distribution of participants according to experience with research-related activities and age group

	Age groups			
	20-34yrs	35-49yrs	50yrs+	Total
Experience	9	17	13	39
No experience	9	8	2	19
Total	18	25	15	59

Gender

Fisher's Exact Probability Test (2-tailed) indicated a non-significant difference in any research-related experience according to gender, *p* = .266.

Table S3 Contingency table displaying distribution of participants according to experience with research-related activities and gender

	Gender		
	Female	Male	Total
Experience	35	5	40
No experience	14	5	10
Total	49	10	59

Professional Discipline

A Fisher's Exact Probability Test (2-tailed) indicated a statistically significant difference between any research-related activity experience and discipline *p* = .048, *phi* = -.28 (low to medium effect size) weighted towards OTs with more experience.

Table S4 Contingency table displaying distribution of participants according to experience with research-related activities and discipline

	Discipline		
	SW	OT	Total
Experience	20	20	40
No experience	15	4	19
Total	35	24	59

Research-related experience (any) and RCC domain comparisons

A Mann-Whitney U Test revealed no significant differences in the participants' overall RCC domain Organisation rating in participants with no research-related experience (*Md* = 4, *n* = 19) and participants with research-related experience (*Md* = 5, *n* = 40), *U* = 269.5, *z* = -1.8, *p* = .07, *r* = .24.

A Mann-Whitney U Test revealed no significant differences in the participants' overall RCC domain Team rating in participants with no research-related experience (*Md* = 3, *n* = 19) and participants with research-related experience (*Md* = 3.8, *n* = 40), *U* = 312.5, *z* = -1.1, *p* = .27, *r* = .14.

A Mann-Whitney U Test revealed a significant difference in the participants' overall RCC domain Individual rating in participants with no research-related experience (*Md* = 2, *n* = 19) and participants with research-related experience (*Md* = 4, *n* = 40), *U* = 256.0, *z* = -2.0, *p* = .04, *r* = .27.

Part 2: Across published studies comparative analyses: workplace settings and discipline

A series of *t*-tests were undertaken to examine the relative impact that professional discipline mix and workplace setting may have on the RCC domains. No test result reached statistical significance.

Table S5 Comparative analyses (independent samples t-tests) across studies: Professional Discipline

Study sample discipline mix		N sample groups	Mean	Std. Deviation	*t*-test	*df*	*p*-value
RCC Organisation	discipline specific	6	97.1	18.0			
	mixed disciplines	7	100.6	15.3	-0.4	11	0.71
RCC Team	discipline specific	6	90.4	20.4			
	mixed disciplines	7	92.1	18.4	-0.2	11	0.88
RCC Individual	discipline specific	6	66.5	17.6			
	mixed disciplines	7	63.8	8.5	0.4	11	0.72

Table S6 Comparative analyses (independent samples t-tests) across studies: Workplace setting

Study sample service/organisation mix		N sample groups	Mean	Std. Deviation	*t*-test	*df*	*p*-value
RCC Organisation	single service/org	6	99.5	15.1	-0.3	9	0.78
	multiple services/orgs	5	102.5	19.5			
RCC Team	single service/org	6	93.1	20.4	0.1	9	0.92
	multiple services/orgs	5	92.0	13.9			
RCC Individual	single service/org	6	64.6	12.6	0.5	9	0.60
	multiple services/orgs	5	60.8	9.6			

One-way ANOVAs were undertaken to examine the relative impact that location of the health service(s) may have had on the RCC domains. No test result reached statistical significance.

Table S7 Abridged RCC summary statistics across published studies partitioned by workplace location

	N sample groups	RCC Organisation *Mean* (*SD*)	RCC Team *Mean* (*SD*)	RCC Individual *Mean* (*SD*)
Metropolitan	4	95.7 (13.2)	83.7 (17.8)	61.0 (13.6)
Regional/rural	3	103.3 (16.3)	106.3 (11.5)	71.2 (6.8)
State/National	4	104.1 (22.3)	91.3 (15.9)	58.5 (9.4)
Total	11	100.8	92.6 (16.9)	62.8 (11.0)

Table S8 Comparative analyses (One-way ANOVA) across published studies examining connections between workplace location and overall RCC domain scores

		Summed of squares	*df*	*F*	*p*-value
RCC organisation	Between groups	168.5	2	0.3	0.77
	Within groups	2523.7	8		
	Total	2692.2			
RCC Team	Between groups	891.7	2	1.8	0.23
	Within groups	1972.8	8		
	Total	2864.5			
RCC Individual	Between groups	297.6	2	1.3	0.32
	Within groups	909.5	8		
	Total	1207.1			
